# Induction of Oxidative Stress in Kidney

**DOI:** 10.1155/2012/465897

**Published:** 2012-04-17

**Authors:** Emin Ozbek

**Affiliations:** Department of Urology, Okmeydani Research & Education Hospital, Sisli, 34384 Istanbul, Turkey

## Abstract

Oxidative stress has a critical role in the pathophysiology of several kidney diseases, and many complications of these diseases are mediated by oxidative stress, oxidative stress-related mediators, and inflammation. Several systemic diseases such as hypertension, diabetes mellitus, and hypercholesterolemia; infection; antibiotics, chemotherapeutics, and radiocontrast agents; and environmental toxins, occupational chemicals, radiation, smoking, as well as alcohol consumption induce oxidative stress in kidney. We searched the literature using PubMed, MEDLINE, and Google scholar with “oxidative stress, reactive oxygen species, oxygen free radicals, kidney, renal injury, nephropathy, nephrotoxicity, and induction”. The literature search included only articles written in English language. Letters or case reports were excluded. Scientific relevance, for clinical studies target populations, and study design, for basic science studies full coverage of main topics, are eligibility criteria for articles used in this paper.

## 1. Introduction

Free radicals are chemical species with a single unpaired electron, which is highly reactive as it seeks to pair with a new free electron, and as a result of these reactions, other free radicals or paired electrons occur and radical feature may be lost. If newly formed free radical occurs, it is also unstable and it can react with another molecule to produce another free radical or a nonradical molecule occurs because of the paired electrons of the newly formed molecule. Thus, a chain reaction of free radicals occurs, leading to damaging biological systems and tissues. In aerobic conditions, all biological systems are exposed to oxidative stress (OS), either generated internally or as by-products. The great majority of these free radicals are mainly oxygen radicals and other reactive oxygen species (ROS) [1].

The well-known ROS are superoxide ion (O_2_•−), hydrogen peroxide (H_2_O_2_), and peroxyl radical (OH•), and the reactive nitrogen species (RNS) are nitric oxide (NO) and peroxynitrite (ONOO−). Peroxynitrite generates from rapid chemical interaction between NO and O_2•_−. Main sites of ROS produced in living organisms are mitochondrial electron transport system, peroxisomal fatty acid, cytochrome P-450, and phagocytic cells [2–6]. Several extracellular and intracellular factors such as hormones, growth factors, proinflammatory cytokines, physical environmental factors (like ultraviolet irradiation), nutrient metabolism, and the detoxification of various xenobiotics affect production of OS [7–13].

In physiological conditions, ROS produced in the course of normal conditions are completely inactivated by cellular and extracellular defence mechanisms. This means that normally there is a balance between prooxidant (or oxidant) and antioxidant defence systems. In certain pathological conditions, increased generation of ROS and/or depletion of antioxidant defence system leads to enhanced ROS activity and OS, resulting tissue damage. OS causes tissue damage by different mechanisms including promoting lipid peroxidation, DNA damage, and protein modification. These processes have been implicated in the pathogenesis of several systemic diseases including kidney.

Several systemic diseases such as hypertension, diabetes mellitus, metabolic syndrome, and hypercholesterolemia; infection; antibiotics and chemotherapeutic agents, and radiocontrast agents mainly excreted from kidney; and environmental toxins especially heavy metals such as lead and mercury, occupational chemicals such as urban fine particles, radiation, smoking, as well as alcohol consumption induce renal OS. The kidney is an organ highly vulnerable to damage caused by ROS, likely due to the abundance of long-chain polyunsaturated fatty acids in the composition of renal lipids. In recent years, OSs have become one of the most popular topics in research of molecular mechanism of renal diseases. The aim of this paper is to summarize the conditions inducing OS in kidney and molecular mechanisms of this induction and kidney damage.

## 2. Diabetes Mellitus and Induction of Oxidative Stress in Kidney

Recent years, diabetes and diabetic kidney disease continue to increase worldwide. In the USA, diabetes-associated kidney disease is a major cause of all new cases of end-stage kidney disease. All diabetic patients are considered to be at risk for nephropathy. Today we have not specific markers to expect development of end-stage renal disease. Clinically control of blood sugar level and blood pressure regulations are important two parameters to the prevention of diabetic nephropathy [14, 15].

There are huge amount of in vitro and in vivo studies regarding explanation of mechanism of diabetes-mellitus-induced nephropathy. All of these mechanisms are a consequence of uncontrolled elevation of blood glucose level. Currently the proposed mechanism is the glomerular hyperfiltration/hypertension hypothesis. According to this hypothesis, diabetes leads to increased glomerular hyperfiltration and a resultant increased glomerular pressure. This increased glomerular pressure leads to damage to glomerular cells and to development of focal and segmental glomerulosclerosis [16, 17]. Angiotensin II inhibitors reduce glomerular pressure and prevent albuminuria. Increased angiotensin II level induces OS through activation of NADPH oxidase, stimulating inflammatory cytokines, and so forth … [18, 19].

The mechanism by which hyperglycemia causes free radical generation thus causes OS to be complex. Increased blood glucose promotes glycosylation of circulator and cellular protein and may initiate a series of autooxidation reactions that culminate in the formation and accumulation of advanced glycosylation end-products (AGEs) in tissues. The AGEs have oxidizing potential and promote tissue damage by oxygen-free radicals [20].

In experimental studies, formation of OS increases because of high level of blood glucose. Sadi et al. showed that in diabetic rat kidney antioxidant enzyme, namely, catalase (CAT) and glutathion peroxidase (GSHPx), activities were found to be reduced; however,  ∝-lipoic acid and vitamin C administration increased these antioxidant enzyme activities [21]. Increased OS is the common finding in tissues effecting from diabetes, including kidney. Reddi et al. showed that transforming growth factor *β*_1_ (TGF-*β*_1_) is prooxidant and Se (selenium) deficiency increases OS via this growth factor. In addition Se deficiency may simulate hyperglycemic conditions. Se supplementation to diabetic rats prevents for formation of OS and renal structural injury [22]. Chen et al. showed that nitrosative stress increases in diabetic rat model [23]. These results show the induction of oxidative and nitrosative stress in rat kidney. These may have a role in pathophysiology of diabetes-induced morphological and functional changes of kidney.

## 3. Hypertension, Hypercholesterolemia, Obesity, and Aging Induce Oxidative Stress in Kidney

Hypertension is one of the major causes of development of renal failure. Key regulator of this pathology is OS. Renal artery stenosis is the most common cause of secondary hypertension and may lead to deterioration of renal function and ischemic nephropathy. Chade et al. showed that a cross-talk between hypoperfusion and atherosclerosis to interactively increased OS, inflammation, and tubular injury in the stenotic kidney [24]. In experimental atherosclerotic renovascular disease (simulated by concurrent hypercholesterolemia and renal artery stenosis), it was reported that the activity of both CuZn and MnSOD isoforms was significantly decreased; however, protein expression of both the NAD (P)H-oxidase subunits p67phox and p47phox, nitrotyrosine, inducible nitric oxide synthase (iNOS), and nuclear factor kappa-B (NF*κ*B) increased. Furthermore, tubular and glomerular protein expression of nitrotyrosine is significantly elevated [25]. Chronic blockade of OS with antioxidant improves OS in kidney. All of these molecular abnormalities suggest increased OS in rat kidney.

Noeman et al. showed that high-fat diet-induced obesity is accompanied by increased hepatic, cardiac, and renal tissue OS, which is characterized by reduction in the antioxidant enzymes activities and glutathione levels, that correlate with the increase in MDA and protein carbonyl (PCO) levels [26]. Increased cytokine release (inflammation-related cytokines such as tumor necrosis factor-∝ and adiponectin) and renal macrophage infiltration have been shown to contribute to renal injury in models of obesity. Chow et al. reported that monocyte chemoattractant protein-1 (MCP-1) is a potent stimulator of macrophage recruitment. It is increased in adipose tissue during obesity and in diabetic kidneys, suggesting that inflammation of these tissues may be MCP-1 dependent [27]. Knight et al. also showed an increase in renal macrophage-specific CD68-positive staining in a model of obesity and hypertension [28]. From these results, we can say that macrophages are the source of increased OS and renal injury in diabetes and obesity-induced renal injury. Aging is associated with increased OS. Most of age-dependent changes in the kidney such as excessive fibrosis, a general lack of regenerative ability, and an increase in apoptosis in cells that determine healthy renal functions are often related to excess OS [29]. At a molecular level, with aging increased mutations in nuclear and mitochondrial DNA (mtDNA), increased lipofuscin and AGEs, increased OS, and increased apoptosis have been observed. Proximal tubular cells contain large numbers of mitochondria and are the most reliant upon oxidative phosphorylation and most susceptible to oxidant-induced apoptosis and mutations [30]. Recent studies showed that antiaging gen, klotho, is important in renal aging and OS-induced renal damage. The *klotho *gene encodes the klotho protein, a single transmembrane protein of the beta-glycosidase family [31]. Mice overexpressing *klotho *exhibit an extension in lifespan and resistance to oxidative injury. Klotho is predominantly expressed in the kidney, with its highest expression in cells of the distal convoluted tubule [32, 33]. Overexpression of *klotho *has been found to enhance resistance to OS through the upregulation of manganese superoxide dismutase (MnSOD). Yamamoto et al. found that klotho modulates MnSOD in a FoxO-dependent process [34]. MnSOD is found within the mitochondria where it acts as primary scavenger of oxidants.

## 4. Urinary Obstruction, Urolithiasis, Infection, Ischemia Reperfusion Injury, Transplantation of Kidney, and Induction of Oxidative Stress in Kidney

Most clinical and experimental studies have shown that OS is increased in kidney and systemic circulation. Huang et al. reported that the activities of catalase and manganese superoxide dismutase were elevated in early stage of ethylene glycol-induced urolithiasis model in rats; however, on day 42 almost all antioxidant enzyme activities were attenuated except those of CAT. In this experiment, the possible mechanism that causes free radical elevation in the kidney may be different in the course of ethylene glycol-induced urolithiasis. Initially systemic circulation may bring the toxic substances to the kidney, and eventually these substances cause to produce free radicals. In the late stage progressive accumulation of leucocytes and defective antioxidant enzyme activities may cause kidney to remain under huge amount of OS [35, 36]. In our experimental urolithiasis studies, we showed decreased antioxidant enzyme activities and involvement of NF*κ*B and p38-MAPK (mitogen-activated protein kinase) signaling pathways, related to OS in rat kidney [37–40]. In in vitro cell culture studies using proximal tubular origin line derived from pig proximal tubules (LLC-PK1), and collecting duct origin Madin-Darby canine kidney (MDCK) cell lines, it has been reported that calcium phosphate crystals cause cellular injury by increasing ROS [41].

Today, extra corporeal shock wave lithotripsy (ESWL) is widely used for the treatment of renal stones in selected renal cases. In our work, we showed increased expression of inducible NO synthase (iNOS) and NF*κ*B, indirect evidence of increased OS [42]. Recently, Gecit et al. showed that ESWL treatment produces OS and causes impairment in the antioxidant and trace element levels in the kidneys of rats [43]. ESWL is associated with greater prevalence of hypertension [44]. Ischemia insult and increased renal OS and consequent endothelial dysfunction may be possible mechanism of hypertension after ESWL.

Urinary obstruction, especially ureteral obstruction due to urolithiasis, is a common urological problem seen in urology practice. Unilateral ureteral obstruction (UUO) leads to decreased renal MnSOD and CAT protein expression in a time-dependent manner. Increased 4-hydroxynoneal (4-HNE) stain for ROS products in the renal tubulointerstitial compartment occurred after 4 hr of UUO in the kidney. The authors explain renal tubular apoptosis in UUO rat model explained by increasing ROS in this study [45]. Various markers of OS increase in UUO rat kidneys such as the oxidatively damaged protein product Ne-carboxymethyl-lysine (CML); the marker of DNA oxidant damage, 8-hydroxy-2′′-deoxyguanosine (8-OHdG)); and lipid peroxidation markers such as malondialdehyde (MDA), 8-iso prostaglandin F2∝  (8-iPGF2∝), and 4-HNE or 4-hydroxy-hexenal (4-HHE). OS response molecules like heat shock protein-70 (HSP-70), heat-shock protein-27, and heme oxygenase-1 (HO-1) [46–51] are strongly expressed after UUO. Mice that are genetically deficient endogenous antioxidant enzyme CAT are more susceptible to UUO-induced renal damage than normal wild type mice. Furthermore, increased renal concentrations of ROS have been observed in obstructed kidneys, together with decreased activities of the major protective antioxidant enzymes SOD, CAT, and glutathione peroxidase [52, 53]. UUO-induced nephrotoxicity and renal fibrosis is thought to be secondary to increased OS in kidney. In the literature there is some information about the amelioration of antioxidant and reactive oxygen scavenger agents against UUO-induced renal damage [54, 55]. For an excellent detailed review, please refer to [56].

Infection is another inducer of OS in kidney. There are a lot of experiments showing the increased oxidative stress and decreased antioxidant defence mechanism, antioxidant enzyme systems in kidney due to infection [57–60].

ROS are important mediators exerting toxic effects on various organs, including kidney during ischemia-reperfusion (IR) injury. A large body of evidences indicate the role of increased OS in the kidney and protective role of antioxidants and ROS scavengers in IR injury-induced nephropathy in the literature [61–63]. OS also has a role as a mediator of injury in chronic allograft tubular atrophy and interstitial fibrosis in rat kidney [64]. Renal transplantation is another OS inducer in kidney in human and animals. OS increases in transplanted kidney because of pretransplant and posttransplant conditions. If there is preexisting diseases such as chronic kidney failure, inflammation, and diabetes mellitus, kidneys are more sensitive to OS during reperfusion injury. Postoperative immunosuppressive agents are among many risk factors inducing OS in kidney [65].

## 5. Antibiotics, Antineoplastic Agents, Immunosuppressants, Analgesics, Nonsteroidal Antiinflammatory Drugs, and Radiocontrast Agents Induce Oxidative Stress in Kidney

Antibiotics, commonly used aminoglycosides, are nephrotoxic agents. Their nephrotoxicity is mainly attributed to induction of OS and depletion of antioxidat enzyme activities in kidney. In our experiments, we showed that iNOS/NF*κ*B/p38MAPK pathway, OS taking place in this axis, is involved in gentamicin-induced nephrotoxicity [66, 67]. Our and other studies showed the protective effect of anti oxidants and reactive oxygen scavenger agents against gentamicin-induced nephrotoxicity [68–70].

Antineoplastic agents are commonly used for the treatment of metastatic cancers. Some of these are nephrotoxic. Excess ROS production and depressed antioxidant defence mechanism are responsible from nephrotoxicity. Cisplatin is the well-known and commonly used antineoplastic and nephrotoxic agent. Other nephrotoxic anticancer agents are carboplatin, methotrexate, doxorubicin, cyclosporine, and adriamycin. Immunosuppressant such as sirolimus and cyclosporine leads to nephrotoxicity via OS [71–80].

Cisplatin is one of the commonly used potent antitumor drugs and cisplatin-based combination protocols are used as front-line therapy for several human malignancies. Cisplatin is toxic to the renal proximal tubules and dose dependent [81, 82]. Several studies have reported the role of OS regarding cisplatin-induced nephrotoxicity. Cisplatin is known to accumulate in mitochondria of renal tubular epithelial cells together with ROS; renal tubular cell mitochondrial dysfunction is also important in cisplatin-induced nephrotoxicity. Mitochondria also continuously scavenge ROS via the action of antioxidant enzymes such as SOD, GSHPx, CAT, and glutathione S-transferase. Studies have demonstrated that cisplatin induces ROS in renal epithelial cells primarily by decreasing the activity of antioxidant enzymes and by depleting intracellular concentrations of GSH. In vitro studies using LLC-PK1 cells, which is characteristic of renal proximal tubular epithelium, also showed the role of ROS in cisplatin nephrotoxicity [83–89].

In this era, analgesics, especially paracetamol and acetaminophen (APAP), and nonsteroidal antiinflammatory drugs (NSAIDs) are widely used throught the world. Paracetamol and APAP are nephrotoxic drugs. Several in vitro and in vivo studies showed that analgesics nephrotoxicity is caused by increased ROS in kidney. Zhao et al. showed the increased ROS, nitric oxide, and MDA levels, together with depleted glutathione (GSH) concentration in the kidney of rats. However, rhein, Chinese herb, can attenuate APAP-induced nephrotoxicity in a dose-dependent manner [90]. We showed in our experiment a significant increase in MDA and decreases in GSHPx, CAT, and SOD activities in APAP-treated rat kidneys. These findings support the induction of OS in rat kidney by APAP. Significant beneficial changes were noted in serum and tissue OS indicators in rats treated with strong antioxidant pineal hormone melatonin and curcumin [91, 92]. Ghosh et al. reported increased OS and TNF-alpha production in rat tissues [93]. Efrati et al. reported that diclofenac (NSAID) leads to nephrotoxicity by increasing intrarenal ROS in rat kidney, and antioxidant, N-acetylcysteine, prevents kidney damage [94].

Contrast-induced nephropathy (CIN) is a major clinical concern, particularly with imaging procedures. CIN is the third most common cause of acute kidney injury in hospitalized patients [95]. Experimental findings in vitro and in vivo illustrate enhanced hypoxia and the formation of ROS within the kidney following the administration of iodinated contrast media, which may play a role in the development of CIN. Studies indeed support this possibility, suggesting a protective effect of ROS scavenging or reduced ROS formation with the administration of N-acetyl cysteine and bicarbonate infusion, respectively [96–99].

## 6. Alcohol, Smoking, Environmental Toxins, Radiation, and Mobile Phones Induce Oxidative Stress in Kidney

Ethanol and its metabolites are excreted into urine, and its content in the urine is higher than that of the blood and the liver. Chronic alcohol administration decreases the renal tubular reabsorption and reduces renal function. Functional abnormalities of renal tubules may be associated with ethanol-induced changes in membrane composition and lipid peroxidation. Because of high content of long-chain-polyunsaturated fatty acids, kidney is highly sensitive to OS damage [100].

Recently it is reported that ethanol administration caused a significant decrease in the levels of antioxidant enzyme CAT, SOD, and GSHPx activities and increases MDA in kidney of the rats [101]. Shankar et al. showed that renal metabolism of ethanol via Cytochrome P450 2E1 (CYP2E1) and antidiuretic hormone-1 led to production of renal OS, and activation of MAPK induces CYP24A1 resulting in reducing circulating 1,25 (OH)2 D3 concentrations [102]. Pathogenesis of aldosterone/salt-induced renal injury similarly is attributed to increased ROS and activation of MAPK in rat kidney [103]. In other studies, authors showed that chronic ethanol administration and cigarette smoke exposure may cause renal injury by increasing oxidative and nitrosative stress in rat kidney [104, 105].

Epidemiological studies have shown that smoking is an accelerating risk factor for the development of nephropathy, in which TGF*β*_1_  plays a role in diabetic patients. Cell culture studies using mesangial cell showed that smoking could increase TGF*β*_1_, probably due to increased oxidative stress and PKC*β*  (protein kinase C beta) activation. This finding supports the concept that smoking is a risk factor for development of diabetic nephropathy by increasing OS in kidney [106]. Similarly smoking and alcohol together increase OS and suppress antioxidant defence mechanism in kidney [104, 105].

In modern era, especially industrialised countries environmental toxins such as air pollutions, substances in stored foods, radiations, as well as heavy metals in waters especially in underdeveloped countries are major health problem. Ochratoxin A (OTA), a mycotoxin, produced by fungi of improperly stored food products, has been linked to the genesis of several disease states in both animals and humans. It has been reported as nephrotoxic, carcinogenic, teratogenic, immunotoxic, and hepatotoxic in laboratory and domestic animals [107–109]. In primary rat kidney cells and in vivo experiments, it has been shown that OTA induces OS and depletes antioxidant systems. These events might represent pivotal factors in the chain of cellular events leading into nephrotoxicity of OTA. Cadmium (Cd) is known to be a widespread environmental contaminant and a potential toxin that may adversely affect public health. Cigarette smoke and food (from contaminated soil and water) are important nonindustrial sources of exposure to Cd. Cd accumulates in the kidney because of its preferential uptake by receptor-mediated endocytosis of freely filtered and metallothionein bound Cd (Cd-MT) in the proximal tubule. Internalised Cd-MT is degraded in endosomes and lysosomes, releasing free Cd into the cytosol, where it can generate ROS and activate cell death pathways [110].

Another environmental nephrotoxic agent is diazinon (O,O-diethyl-O-[2-isopropyl-6-methyl-4-pyrimidinyl] phosphorothioate). It is an organophosphate insecticide and has been used worldwide in agriculture and domestic for several years. Shah et al. showed that diazinon exposure depletes antioxidant enzymes and induces OS in rat kidney [111].

Increased air pollution as a result of industry is another life threatening health problem. Boor et al. showed renal, vascular, and cardiac fibrosis in rats exposed to passive smoking and industrial dust fibre amosite. Authors explain these changes by increased OS in these tissues [112]. Nanosized fraction of particulate air pollutions have been reported to translocate from the airways into the bloodstream and act on different organs such as lungs, heart, liver, and kidneys. Nemmar et al. examined the distribution and the pathological changes of diesel exhaust particles (DEPs) on systolic blood pressure (SBP), systemic inflammation, oxidative stress, and morphological alterations in lungs, heart, liver, and kidneys in Wistar rats. They showed that DEPs cause inflammation especially in lungs and pulmonary tissue, and these pathological changes are attributed to increased OS and inflammatory cytokines in these tissues [113]. Lead and cadmium nephrotoxicity are also related to increased OS in kidney [114, 115].

Radiation is an important inducer of OS. For diagnostic and therapeutic purposes, radiation is commonly used. Chronic OS after total body irradiation is thought to be the cause of radiation nephropathy in rats. Authors looked for evidence of OS after total-body irradiation in a rat model; focusing on the period before that there is physiologically significant renal damage. No statistically significant increase in urinary 8-isoprostane (a marker of lipid peroxidation) or carbonylated proteins (a marker of protein oxidation) was found over the first 42 days after irradiation, while a small but statistically significant increase in urinary 8-hydroxydeoxy-guanosine (a marker of DNA oxidation) was detected at 35–55 days. In renal tissues, they found no significant increase in either DNA or protein oxidation products over the first 89 days after irradiation. They suggest that if chronic OS is a part of the pathogenesis of radiation nephropathy, it does not leave widespread or easily detectable evidence behind. Emre et al. investigated the effect of extremely low-frequency electromagnetic field (ELF-EMF) with pulse trains exposure on lipid peroxidation and hence oxidative stress in the rat liver and kidney tissue. They found increases in the levels of oxidative stress indicators, and the flow cytometric data suggested a possible relationship between the exposure to magnetic field and the cell death; however, there were significantly lower necrotic cell percentages in experimental animals compared to either unexposed or sham control groups [116]. These results suggest the inductive effect of radiation on OS in kidney.

For the last two decades, a large number of studies have investigated the effects of mobile phone radiation on the human and animal. Cellular target and tissue damage are different. Male reproductive system is among the most affected system [117, 118]. Increased OS plays a central role in radiofrequency-electromagnetic-waves- (RF-EMW-) induced tissue damage. Devrim et al. examined the effect of RF-EMW on oxidant and antioxidant status in erythrocytes and kidney, heart, liver, and ovary tissues from rats and possible protective role of vitamin C. It was observed that MDA level, xanthine oxydase (XO), and GSH-Px activities significantly increased in the EMR group as compared with those of the control group in the erythrocytes. In the kidney tissues, it was found that MDA level and CAT activity significantly increased, whereas XO and adenosine deaminase (ADA) activities decreased in the cellular phone group as compared with those of the control group. However, in the heart tissues, it was observed that MDA level, ADA, and XO activities significantly decreased in the cellular phone group as compared with those of the control group. They concluded that RF-EMR at the frequency generated by a cell phone causes OS and peroxidation in the erythrocytes and kidney tissues from rats. In the erythrocytes, vitamin C seems to make partial protection against the OS [119]. Ozguner et al. reported similar results in rat experiments and they also reported that preventive effect of Caffeic acid phenethyl ester (CAPE), a flavonoid-like compound, is one of the major components of honeybee propolis and melatonin against RF-EMW-induced nephrotoxicity [120–122].

## 7. Conclusion

There is huge amount of literature concerning the link between the OS and renal diseases. Systemic diseases such as hypertension, diabetes mellitus, and hypercholesterolemia; infection; antibiotics, chemotherapeutics, and radiocontrast agents; and environmental toxins, occupational chemicals, radiation, smoking, as well as alcohol consumption induce renal OS. The kidney is a highly vulnerable organ to damage caused by ROS, due to the abundance of long-chain-polyunsaturated fatty acids. Antioxidant and reactive oxygen scavengers have been shown to be effective in animals for protecting kidney, but it is hard to translocate these results to humans. This may be due to short duration of animal studies, dose differences between animals and humans, and different pathophysiologic processes between animals and humans. For understanding these steps, drugs will be developed to alter the main process(es) responsible for increased OS. In this paper, the conditions inducing OS in kidney and molecular mechanisms of this induction and kidney damage have been summarized. I hope that this paper will aid in the understanding of this complex system and directing new research effort.
